# A Proposal of a Practical and Optimal Prophylactic Strategy for Peritoneal Recurrence

**DOI:** 10.1155/2012/340380

**Published:** 2012-02-08

**Authors:** Masafumi Kuramoto, Shinya Shimada, Satoshi Ikeshima, Akinobu Matsuo, Hiroshi Kuhara, Kojiro Eto, Hideo Baba

**Affiliations:** ^1^Department of Surgery, Yatsushiro Social Insurance General Hospital, 2-26 Matsuejo, Yatsushiro, Kumamoto 866-8660, Japan; ^2^Department of Gastroenterological Surgery, Graduate School of Medical Sciences, Kumamoto University, Honjo 1-1-1, Kumamoto 860-8556, Japan

## Abstract

Peritoneal metastasis, which often arises in patients with advanced gastric cancer, is well known as a miserable and ill-fated disease. Once peritoneal metastasis is formed, it is extremely difficult to defeat. We advocated EIPL (extensive intraoperative peritoneal lavage) as a useful and practical adjuvant surgical technique for those gastric cancer patients who are likely to suffer from peritoneal recurrence. In this paper, we review the effect of EIPL therapy on prevention of peritoneal recurrence on patients with peritoneal free cancer cells without overt peritoneal metastasis (CY+/P−) through the prospective randomized study, and we verified its potential as an optimal and standard prophylactic therapeutic strategy for peritoneal recurrence.

## 1. Introduction

Significant advances in surgical technique and perioperative management have dramatically improved the survival of patients with advanced gastric cancer; nevertheless, peritoneal metastasis is still the most common cause of tumor progression, and the prognosis of those patients with peritoneal recurrence remains extremely poor [1–5]. The median survival time (MST) of such patients is reported to be 3–6 months [6], and a standard regimen against peritoneal metastasis of gastric cancer has not yet been established [7–10].

In patients with serosal invasion, about half develop peritoneal recurrence and die of this disease during the first 2 years of followup, even if curative resection is performed [11, 12]. Further, it has been reported that the survival time of patients with cytology-positive peritoneal lavage fluid and without macroscopic peritoneal dissemination (CY+/P−) of gastric cancer was almost the same as that of patients with overt peritoneal metastasis [13], and the 5-year survival rate of patients with CY+/P− is only 2% [14]. Once peritoneal metastasis develops, it is quite impossible for patients to survive. The results of several randomized clinical trials which have been published before on perioperative intraperitoneal chemotherapy for patients with CY+/P− or peritoneal metastasis have not shown any significant demonstrations of improvement in survival as compared with surgery alone, especially in patients with peritoneal metastasis [3, 8, 15–18].

It is already generally accepted that peritoneal metastasis is completed by the implantation of peritoneal free cancer cells exfoliated from serosa-invasive tumors. Consequently, it is considered important to prevent peritoneal metastasis before the fixation and progression of free cancer cells on the peritoneum of patients with advanced gastric cancer. This is because the presence of intraperitoneal free cancer cells without macroscopic dissemination could possibly mean a condition where the implantation of cancer cells on the peritoneal wall has not yet occurred. The situation of CY+/P− might be the last opportunity for surgeons to undertake surgical intervention to rescue such patients, and therefore, a reliable and appropriate standard prophylactic treatment needs to be established to prevent CY+/P− gastric cancer developing into peritoneal metastasis.

From this point of view, we have been advocating the adoption of “extensive intraoperative peritoneal lavage” (EIPL) as a useful intraoperative technique for an adjuvant therapy to avoid the implantation of cancer cells on the intraperitoneal wall after a potentially curative resection, combined with intraperitoneal chemotherapy (EIPL-IPC). EIPL is very simple and can be performed anywhere and anytime. Also, it has quite an amazing power of reducing the number of intraperitoneal free cancer cells efficiently to potentially zero, analyzed by a detection system of cancer cells using real-time reverse transcriptase-polymerase chain reaction (RT-PCR), and intraperitoneal chemotherapy subsequent to EIPL could play an important role in eradicating any remaining cancer cells. We have confirmed the clinical effectiveness of EIPL by ultrarapid quantitative RT-PCR protocol. Quite a few intraperitoneal free cancer cells could be detected in the washing lavage fluid after 6 to 8 washes. Finally, our recent prospective randomized control study clearly revealed that EIPL-IPC therapy significantly improved the 5-year survival of advanced gastric cancer patients with CY+/P− [19].

In this article, we reviewed the efficacy and advantage of our new adjuvant intraoperative method to reduce the peritoneal recurrence, and clarified the feasibility and validity of adopting this method as the standard prophylactic strategy for the prevention of peritoneal metastasis in advanced gastric cancer patients.

## 2. Conventional Treatment of CY+ Gastric Cancer

To date, many studies on positive intraperitoneal free cancer cells (CY+) in patients with advanced gastric cancer without overt peritoneal metastasis have been conducted to assess whether CY+ could be a predictive factor. Although most of the studies succeeded in showing the validity of CY+ as a reliable predictive factor, there are not yet any reports concerning drastic and effective therapies for patients with CY+ [20–27]. As mentioned already, the simple existence of free cancer cells in the peritoneal cavity is apparently different from that of peritoneal dissemination; moreover, the status of CY+/P− includes the condition where peritoneal metastasis has not yet occurred. So, we have been focusing on devising a beneficial method that could improve the survival of CY+ patients surgically.

## 3. EIPL (Extensive Intraoperative Peritoneal Lavage)

**Figure 1:**
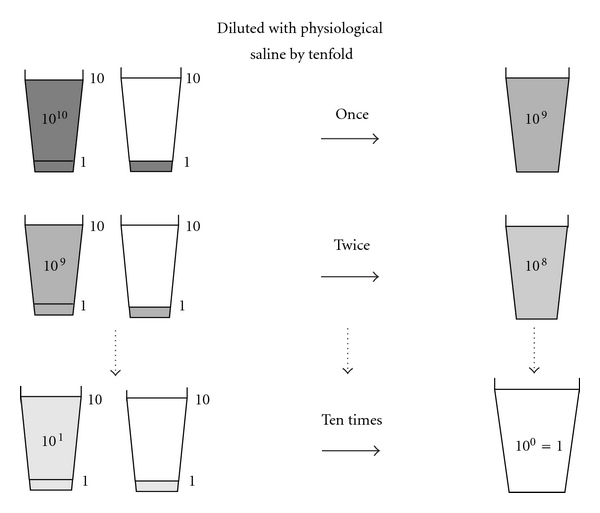
Schema of “limiting dilution method.” This method is expected to lead to a logarithmic reduction of numerous cancer cells to zero.

**Figure 2:**
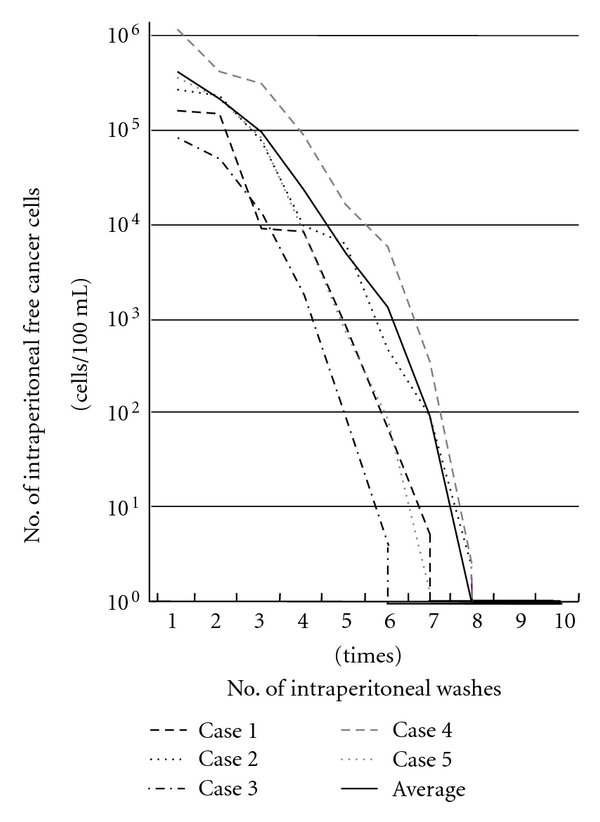
Changes in numbers of intraperitoneal free cancer cells in five gastric cancer patients with CY+ treated by EIPL therapy. The numbers of free cancer cells in 100 mL of samples from the lavage fluid using 1 liter of saline were measured by ulra-rapid RT-PCR. The free cancer cells were serially diluted by 6 to 8 liters of saline and disappeared in washing fluid after that.

We have proposed that EIPL is a quite formidable method for reducing the number of intraperitoneal free cancer cells to potentially zero, just like the so-called “limiting dilution” approach [28]. Briefly, the peritoneal cavity is extensively stirred and washed after the potentially curative operation, which is followed by the complete aspiration of the fluid. This procedure is done 10 times using 1 L of physiological saline. 10 washes of a 1 : 10 dilution result in just 1 cancerous cell from 10^10^ cells in the abdominal cavity (Figure 1). Furthermore, sufficient stirring and washing of the abdominal cavity would remove the cancer cells which merely adhere to the peritoneum. EIPL was performed in five cases of serosa-invasive gastric cancer with CY+/P−, and its efficacy was evaluated by the ultrarapid quantitative RT-PCR protocol, which made it possible to detect mRNA of CEA and CK20 intraoperatively by performing all steps of the procedure in only about 70 minutes. Sequential washing of intraperitoneal free cancer cells of 3.8 ×  10^5^ ± 1.4  × 10^5^/100 mL of lavage decreased the number to 2.8 ± 1.5 cells by 6 to 8 washes. Free cancer cells were not detected in the fluid after that (Figure 2). On the other hand, 2.8 ×  10^4^ ± 4.5  × 10^4^ of intraperitoneal free cancer cells still remained in 100 mL of the lavage when not treated with EIPL. Our preliminary subset analysis based on 22 consecutive patients with CY+/P− who underwent curative surgical treatment for advanced gastric cancer, and who were followed up for 2 years or until death, has shown a statistically significant improvement of a 2-year survival rate when treated with EIPL as compared with when not treated with EIPL [29].

## 4. Clinical Adoption of EIPL-IPC Therapy

Based on our pioneering study, we have advocated EIPL-IPC (intraperitoneal chemotherapy) therapy. After the EIPL treatment, cisplatin (CDDP) is administrated into the abdominal cavity at a dose of 100 mg/body and the solution is drained 1 hour after the injection. In this way, even if only a few cancer cells were to remain, these cells might find it difficult to survive and/or to disseminate due to the effects of IPC.

To clarify the distinct survival effects of EIPL-IPC therapy, we designed a prospective randomized multicenter trial for advanced gastric cancer patients with CY+/P−.

A total of 88 gastric cancer patients with CY+/P− from 1522 patients with advanced gastric cancer at multicenters were enrolled in this study, and were randomly allocated to three groups: surgery alone group, surgery plus intraperitoneal chemotherapy (IPC) group, and surgery plus EIPL and IPC (EIPL-IPC) group. Peritoneal lavage for the surgery alone group and the IPC group was done with 3 liters of saline (1 liter, three times) before the closure of the abdominal wall or IPC, respectively.

**Figure 3:**
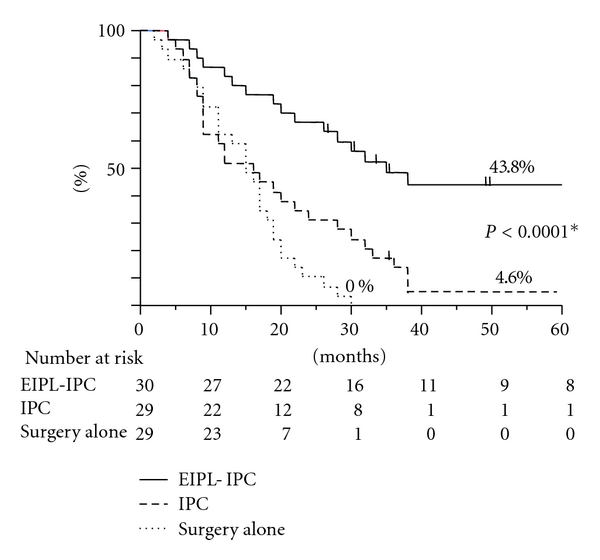
The survival curves for the 88 patients stratified according to the treatments. *By log-rank test.

The overall 5-year survival rate of patients with EIPL-IPC was 43.8%, and this data was significantly higher than that of the IPC group (4.6%, *P* < 0.0001) and the surgery alone group (0%, *P* < 0.0001), as shown in Figure 3. 

 Among various recurrent patterns, the EIPL-IPC group had a significantly lower incidence of peritoneal recurrence than either of the other groups. Univariate and multivariate analyses clearly revealed that EIPL was the most significant impact factor.

The results of this study far exceeded our expectations and showed a remarkably better prognosis than previous studies on gastric cancer patients with CY+/P−. For example, a study on the median survival time (MST) of 91 patients with CY+/P− who had potentially curative operations stated survival to be only 386 days [30], and the 5-year overall survival rate has been 13% [31]. In our study, the surgery alone group as well as the IPC group also showed similar results to the reports just cited. Surprisingly, however, in the EIPL-IPC group the overall 5-year survival rate and MST were 42.1% and 35 months, respectively, remarkably significant improvement of both survival and MST. These results were convincing and are promising enough to serve as a solid basis on which to build strong confidence in and high expectations for employing the EIPL-IPC therapy.

## 5. Further Application of EIPL Therapy

### 5.1. Application to CY−/P− Gastric Cancer

Despite neither the apparent existence of abdominal free cancer cells nor overt peritoneal metastasis, approximately half of patients with serosa-involved gastric cancer developed peritoneal recurrence after curative operations [27]. In addition, some nonserosa-involved gastric cancers advance to peritoneal recurrence, even though a curative operation has been performed [5, 32–34]. We elucidated the mechanisms of peritoneal recurrence after curative operations for patients with nonserosa-involved gastric cancer.

CEA and CK20 mRNA in the peritoneal lavage samples from 63 patients with nonserosa-involved gastric cancer which were obtained just after laparotomy and after lymph node dissection were examined by an ultrarapid quantitative RT-PCR system [28]. In the peritoneal lavage samples from nonserosa-involved cases after lymph node dissection, CEA or CA20 mRNA were detected in 16 of 63 patients (25.4%) despite no detection of either CEA or CA20 mRNA just after laparotomy. These were not evident in the mucosal (M) tumor, but were detected in three (14.3%), six (46.2%), and seven (53.8%) patients with submucosal (SM), muscularis propria (MP), and subserosal (SS) tumors, respectively. These data suggested the existence of free cancer cells in the peritoneal cavity after lymph node dissection with non-serosa-involved gastric cancer patients. Moreover, our previous study on 1272 gastric cancer patients revealed that 1/257 cases (0.4%) of SM and 6/136 cases (4.4%) of MP developed peritoneal recurrences after potentially curative resections [34]. Among them, 86% of the patients had lymph node metastasis and/or lymphatic invasion. Our results demonstrated that lymph node dissection would be a main factor for spreading viable free cancer cells into the peritoneal cavity. Thus, we came to an assurance that lymph node dissection itself is a cause of peritoneal dissemination, seeding viable cancer cells from the lymphatic vessels to the abdominal cavity. As there should be a low risk of the completion of peritoneal metastasis in such cases with non-serosal-involved gastric cancer, EIPL therapy will demonstrate its effectiveness to the maximum on the prevention of peritoneal recurrences after curative operations.

### 5.2. Application to Other Abdominal Organ Cancers

We applied EIPL therapy to the miserable disease of pancreatic cancer, where peritoneal recurrence is frequently found and yields a high mortality rate [35]. EIPL therapy was performed consecutively on 15 patients of 39 patients with invasive ductal adenocarcinoma of the pancreas who underwent curative surgical treatment. The peritoneal recurrence rate of the EIPL group was significantly lower than that of the non-EIPL group (6.7% versus 45.8%, *P* = 0.013) and the EIPL therapy was the independent negative risk factor for peritoneal recurrence. On the basis of such attractive data, EIPL therapy is considered to be applicable to various abdominal cancers which are likely to seed in abdominal cavities.

## 6. Proposal of EIPL Therapy as a Standard Therapeutic Strategy for Prevention of Peritoneal Recurrence

Lymphatic and peritoneal metastasis is well known to be high in advanced gastric cancer while hematogenous metastasis is relatively low [1, 36, 37]. Above all, peritoneal metastasis is the most common cause of tumor progression and death even if curative surgery is performed [1–5]. Once peritoneal metastasis is formed, it becomes extremely difficult for patients to survive through to a cure, though the survival time has become somewhat longer by excellent means including intravenous and/or intraperitoneal chemotherapy. Several studies have suggested that chemotherapy could possibly result in much better prognosis than would be expected from aggressive surgery for gastric cancer patients with CY+/P−, just like patients with peritoneal metastasis [20, 21, 38, 39]. On the other hand, there is a report which has demonstrated that radical surgery as well as adjuvant chemotherapy should be performed for CY+/P− patients in cases of no lymph node metastasis [40]. This demonstrates that appropriate standard regimens for patients who are likely to progress toward peritoneal metastasis, including CY+/P− patients, has not yet been established.

As already mentioned, the situation of CY+/P− means the condition where the implantation of free cancer cells derived from the primary tumor has not yet occurred. We suppose there should be apparent differences between the conditions of CY+/P− and peritoneal metastasis which would require different management strategy. Therefore, it is considered reasonable and relevant to focus on devising some effective surgical measures to prevent peritoneal recurrence, accompanied by appropriate and respectable radical resection. Although the Dutch report has described the high postoperative morbidity and mortality after gastrectomy with D2 lymph node dissection [41], radical resections with D2 lymphadenectomy appear to be feasible and safe for patients in Japan [20, 21, 38, 39]. In our study, operative morbidity and mortality were 1.5% and 0.5%, respectively. These results show that potential benefits of D2 operations would outweigh the risk of morbidity and mortality after the radical operation. Complete extirpation of gastric cancer with a sufficient resection margin from the tumor and removal of metastatic lymph nodes is the only measure that could bring the hope of cure for patients with gastric cancer [1, 36, 37, 42, 43], therefore, advanced gastric cancer should be treated with radical resection even if it is accompanied by CY+/P− because our novel EIPL-IPC regimen would have the power to cancel the CY+ condition.

**Figure 4:**
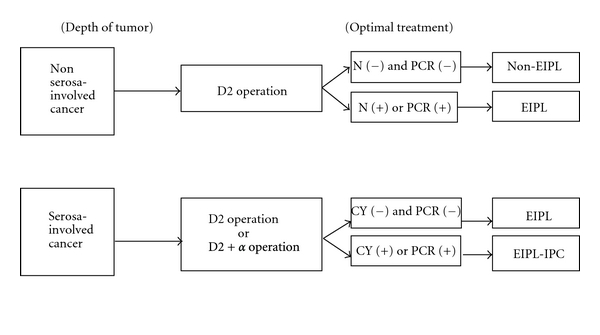
A practical and optimal treatment protocol for advanced gastric cancer. D2 operation: gastrectomy with dissection of group 1 and 2 lymph node [37], N(+): positive lymph node metastasis through operation, N(−): no evidence of lymph node metastasis, PCR: real-time reverse transcriptase-polymerase chain reaction, EIPL: extensive intraoperative peritoneal lavage, IPC: intraperitoneal chemotherapy.

Lastly, we strongly advocate the adoption of the new treatment protocol for advanced gastric cancer as shown in Figure 4. In case of positive lymph node metastasis through operation or positive molecular detections of CEA and CA20 mRNA in the lavage fluid after lymph node dissection, EIPL is performed even for the patients with non-serosa-involved cancer. Except for overt peritoneal metastasis, all patients with serosa-involved cancer undergo EIPL in principle and IPC therapy is added to the patients with CY+ or PCR (+) in the lavage fluid just after laparotomy. After the proper tumor resection and lymphadenectomy, EIPL (or EIPL-IPC) therapy serves an extremely important role for gastric cancer patients with high peritoneal recurrence risk such as serosal invasion and lymph node metastasis. The innovative EIPL-IPC therapy is very practical and its theoretical basis creates high expectations as to the effects of cytoreduction, potentially to zero. Furthermore, EIPL therapy is simple, not time-consuming, inexpensive, and it is not curtailed by place or time, so it can easily be performed anytime, anywhere. Also, it does not require the use of any special techniques or devices. In addition, a point worthy of special mention is that EIPL itself only has minimal risk for patients.

## 7. Conclusion

In conclusion, we reviewed clinical studies concerning EIPL therapy, and very favorable results convinced us to advocate EIPL therapy as an optimal treatment protocol for advanced gastric cancer patients. It is our fervent wish that EIPL therapy be adopted as the standard prophylactic strategy for peritoneal recurrence.
